# The immune checkpoint VISTA exhibits high expression levels in human gliomas and associates with a poor prognosis

**DOI:** 10.1038/s41598-021-00835-0

**Published:** 2021-11-02

**Authors:** Amina Ghouzlani, Abdelhakim Lakhdar, Soumaya Rafii, Mehdi Karkouri, Abdallah Badou

**Affiliations:** 1 Cellular and Molecular Pathology Laboratory, Faculty of Medicine and Pharmacy, Hassan II University, Casablanca, Morocco; 2 Department of Neurosurgery, UHC Ibn Rochd, Casablanca, Morocco; 3 Laboratory of Research on Neurologic, Neurosensorial Diseases and Handicap, Faculty of Medicine and Pharmacy, Hassan II University, Casablanca, Morocco; 4 Department of Pathology, CHU Ibn Rochd, Casablanca, Morocco

**Keywords:** Cancer, Cell biology, Immunology, Molecular biology, Neuroscience, Diseases, Molecular medicine, Neurology, Oncology

## Abstract

In human gliomas, anti-tumor T cell responses are inhibited through induction of local and systemic immunosuppression. Immune checkpoint blockade is proving to be a success in several types of cancers. However, many studies reported that the treatment of glioblastoma patients with anti-CTLA-4 or anti-PD-1 has no survival benefit compared to standard chemotherapy. This study aimed to investigate the expression and role of VISTA, a newly described immune checkpoint regulator, in human gliomas. mRNA expression was assessed in a total of 87 samples from glioma patients. 57 glioma tissues were taken at different grades. 20 peripheral blood mononuclear cells (PBMC) samples were taken before surgery and ten after surgery, all from the same set of patients. As for the control, ten specimens of PBMC were taken from healthy donors. Protein expression using immunohistochemistry was performed for 30 patients. The Cancer Genome Atlas (TCGA) data set, was also used to investigate *VISTA* expression through analysis of RNA-seq data of 667 glioma patients. In the Moroccan cohort, *VISTA* gene expression was significantly upregulated in glioma tissues related to PBMC of healthy donors. This high expression was specific to patient tissues since *VISTA* expression in PBMC was low when assessed either before or after surgery. Besides, VISTA exhibited higher expression levels in grade III/IV relative to grade I/II glioma patients. Interestingly, *VISTA* correlated positively with *PD-1* expression. *PD-1* also showed elevated expressions in higher glioma grades. The TCGA cohort corroborated these observations. Indeed, *VISTA* was also found to be strongly expressed in high grades. It was positively correlated with other critical immune checkpoints. Finally, increased *VISTA* transcript levels were associated with weak overall survival of glioma patients. Our study highlighted a correlation between high levels of VISTA expression and poor prognosis in glioma patients. VISTA might be involved in glioma progression and could be considered as a possible new therapeutic target, especially in advanced gliomas.

## Introduction

Gliomas are the most frequent and violent primary brain tumors in adults^1^. Among glioma types, glioblastoma (GBM) is the most common and invasive type. Despite the multimodal-conventional therapy such as neurosurgical resection and radical or chemotherapy, this pathology remains a significant cause of death in human cancer, with a median survival of only 14.6 months^2^. For a decade, several studies on molecular markers and targeted drugs, generated restricted effect in extending life of glioma patients. The discovery of intracranial lymphatic system has brought a new theoretical basis and a new hope for brain tumor immunotherapy^3^.

In the past few years, a collection of data has clarified the crucial role of the immune checkpoints in regulating the immune response in different cancer types. However, research on immunotherapy of glioma has extended in an exponential manner^4–7^. The majority of gliomas is obstinate to usual immunotherapies. Most of glioma patients did not respond to the blockade of habitual immune checkpoints pathways (CTLA-4 and PD1/PD-L1)^8–10^. This has heightened our interest in finding novel immune checkpoints whose targeting could be beneficial for glioma patients.

Programmed Death 1 (*PD-1*) protein is a co-inhibitory receptor which is expressed on activated T cells, B cells, macrophages, dendritic cells (DC) and monocytes^11,12^. It has been demonstrated that *PD-1* inhibits adaptive and innate immune responses when coupling to its ligands *PDL-1* and *PD-L2*, which are expressed mainly by tumor cells^11,13^. *PD-L1* can block the cytolytic activity of *PD-1*+ tumor-infiltrating *CD4*+ and *CD8*+ T cells and cytokine production^12^. Besides, the latest clinical trials showed that neutralizing monoclonal antibodies (mAbs) against *PD-1* or *PD-L1* resulted in an impressive anti-tumor effect in various types of solid tumors with complete regression in some patients^14^.

V-domain Immunoglobulin suppressor of T cell activation (*VISTA*) is a new Immunoglobulin (Ig) superfamily ligand, which was recently discovered^15^. *VISTA* (also known as c10orf54 or PD-1H) shares sequence homology with *PD-1* and *PD-L1* and can act as a receptor on T lymphocytes or a ligand on antigen-presenting cells^16^.

VISTA expression is observed mostly in most immune cells, including *CD4*+ and *CD8*+ T cells, NK cells, macrophages, DCs and neutrophils, but not B cells. However, *VISTA*-knockout (KO) mice developed a spontaneous accumulation of activated T cells in multiple organs^17^. Also, *VISTA*–KO mice are resistant to the growth of GL261 glioma^18^.

Here, we reported a significant expression profile of VISTA in advanced versus primary glioma grades. VISTA, whose expression correlated with CD8 T cell presence in glioma patients, appeared to be one of the most highly expressed immune checkpoints in this tumor microenvironment. Interestingly, it was found that high VISTA expression is associated with poor patient’s outcome, which strongly suggests that VISTA could be considered as a new potential therapeutic target in advanced gliomas.

## Materials and methods

### Patients and samples

mRNA expression was assessed in a total of 87 samples from glioma patients. There were 57 glioma tissues at different grades:

- 24 grade IV (Glioblastoma), 6 grade III (3 Astrocytomas and 3 Ependymomas), 9 grade II (1 Astrocytoma, 4 Ependymomas, 3 Oligodendrogliomas and 1 Xantoastrocytoma), 18 grade I (16 Astrocytomas and 2 Gangliogliomas).
- 20 peripheral blood mononuclear cell (PBMC) samples taken before surgery and 10 (PBMC) after surgery, all from the same set of patients, at the Ibn Rochd University Hospital, neurosurgery department (Casablanca, Morocco). As for the control, ten specimens of PBMC were taken from healthy donors, at Regional Blood Transfusion Center (Casablanca, Morocco). The inclusion criteria were adopted as follows: informed consent to participate in the study protocol, full documentation of the study. Patients had been previously diagnosed with glioma.

Selected patients have been already diagnosed with glioma and not been undergone any therapy before tumor resection. However, the exclusion criteria were: incomplete documentation of the study, no informed consent available to participate in the study protocol.

The samples were recruited from May 2016 until June 2019. All glioma tissues were graded according to the World Health Organization (WHO) 2007 and 2016, clinical information, including gender, age and smoking status was obtained from the medical records of the patients.

### TCGA data analysis

The RNA-seq and clinicopathological characteristics data from 667 glioma samples were collected from The Cancer Genome Atlas (TCGA) dataset, graded according to the World Health Organization (WHO) from grade II to grade IV, were analyzed in our study (http://cancergenome.nih.gov). The Inclusion criteria adopted were full RNA-seq and clinicopathological characteristics information of each sample. The exclusion criteria were the lack of RNA-seq and clinicopathological characteristics information.

To confirm the generated results, data analysis and statistical tests were carried out by two different scientists in the lab. Gene expression profiling data were log- converted before data analysis.

### Peripheral blood mononuclear cell isolation

Mononuclear cells were isolated by density gradient centrifugation according to Ficoll’s protocol as previously described^19^. 5 ml of human peripheral blood was first mixed with 5 ml of saline solution (0.9% NaCl). This mixture was then added to 5 ml of Ficoll medium (Biowest, France). The total was then centrifugated at 350*g* for 10 min. The layer corresponding to mononuclear cells was collected and washed twice in 0.9% NaCl.

### Total RNA isolation and reverse transcription (RT)

Total RNA was extracted from PBMCs, and frozen glioma samples using TRIzol reagent (Invitrogen, France) as previously described^20^. RNA concentration and quality were measured using the NanoVueTM Plus Spectrophotometer (GE Healthcare, UK). According to the manufacturer’s instructions, cDNA first was synthesized using Tetro Reverse Transcriptase Enzyme (Bioline, France) from 0.5 μg of total RNA in a 20 μl reaction mixture with 1 μl Random Hexamer Primer 25 µg (Bioline, France) and 4 μl of RNase-Free Water added and incubated at 70 °C for 5 min to break the secondary structure of RNA. Then, the mixture was maintained on ice. 4 μl Tetro Reverse Transcriptase buffer, 4 μl of dNTP (10 mM), 0.5 μl of RNase Inhibitor (Invitrogen, France), 0.5 μl Tetro Reverse Transcriptase Enzyme (Bioline, France) and 1 μl of RNase-Free Water were added and incubated at 25 °C for 10 min, then at 45 °C for 30 min and then at 85 °C for 5 min.

### Real-time RT-PCR assays

Relative quantification of gene expression was analyzed by real-time PCR in the presence of the fluorescent dye SYBR Green PCR Master Mix (Thermo Fischer). *β*-Actin was used as an internal control to evaluate the relative expression of *VISTA* and *PD-1*. Experiments were performed in a 20 μl reaction volume with specific primer pairs used at 10 µM for all genes.

PCR was programmed as follows: 10 min at 95 °C for polymerase activation and sample denaturation, then 40 cycles of 15 s at 95 °C and 60 s at 60 °C for annealing and extension. Fluorescence readings, at the end of the extension phase of each cycle, were used to estimate the values for the threshold cycles (Ct). The Ct values for each gene were converted into relative quantification (2^−ΔCt^).

Primer pairs were as follows:

- ***β*-Actin** Forward: 5′-TGGAATCCTGTGGCATCCATGAAAC-3′.
  Reverse: 5′-TAAAACGCAGCTCAGTAACAGTCCG-3′.
- ***VISTA*** Forward: 5-TGTAGACCAGGAGCAGGATG-3′.
  Reverse: 5-ATGCACCATCCAACTGTGTG-3′.
- ***PD-1*** Forward: 5′-GCTGGATTTCCAGTGGCGA-3′.
  Reverse: 5′-ATGAGGTGCCCATTCCGCTA-3′.

### Immunohistochemistry assays (IHC)

30 paraffin-embedded human glioma tissues (13 low grade and 17 high-grade cases) were sectioned (thickness of 3–4 µm). First, samples were incubated at 65 °C for 1 h and then at 37 °C overnight before being deparaffinized and rehydrated. For antigen retrieval step the water bath method was performed, using PT Link (Dako, Denmark) and a low pH (pH = 6) retrieval solution (EnVision Flex target retrieval solution low PH (× 50) 30 ml, Dako, Denmark) at 98 °C for 20 min.

For blocking the endogenous peroxidase activity, samples were immersed in 3% hydrogen peroxide (EnVision flex peroxidase-blocking reagent, Dako, Denmark) for 10 min at room temperature followed by incubation in wash buffer (EnVision flex wash buffer, Dako, Denmark). Two times for 2 min each to reduce non-specific binding. Slides then were incubated with a primary polyclonal rabbit anti-human VISTA antibody at 2 μg/ml (MyBioSource, San Diego, CA, USA). For each case, a second slide was used as a negative control with Rabbit IgG Isotype Control at 1:200 dilution (Clinisciences, France) at room temperature for 45 min.

After rinsing in wash buffer two times for 2 min for each, slides were incubated with a secondary horseradish peroxidase-conjugated goat anti-rabbit anti-mouse IgG (EnVision Flex/HRP, Dako, USA) for 20 min at room temperature. Slides were then rinsed thoroughly in wash buffer, two times for 2 min each, before incubation with diaminobenzidine solution (EnVision DAB + CHROMOGEN, Dako, USA) to develop color for 10 min at room temperature. Finally, slides were counterstained with hematoxylin solution at room temperature for 1 min dehydrated and then mounted to being examined under an Olympus light microscope (Olympus, Tokyo, Japan).

### Evaluation of immunostaining

Intensity, percentage, the intracellular distribution of stained tumor cells (TC), the amount of positive immune cells and positivity of endothelial cells were evaluated separately by the pathologist. The intensity of immunostaining of tumor cells was graded as negative (0), weak (1+), moderate (2+) or strong (3+). VISTA-positive immune cells were counted in intratumoralhotspot regions. Immunostaining of endothelial and immune cells was graded as present or absent^21^.

### Ethics approval and consent to participate

The current study was approved by the Ethical Board of the Ibn Rochd University Hospital of Casablanca. Written informed consent was obtained from glioma patients (from parents and/or legal guardian for the subject under 18 years old), and healthy donors involved in this study. Methods were carried out by relevant guidelines and regulations.

### Statistical analysis

In this study, statistical analysis was performedusing GraphPad Prism 6.0 (GraphPad Software, Inc., La Jolla, CA, USA) or R (version 3.3.1, Auckland, NZ).

The paired t-test was used for paired samples to make a statistical comparison of gene expression between groups. The Mann–Whitney–Wilcoxon test were used for ranked data as appropriate. The two-sided p value less than 0.05 was considered as statistically significant for all statistical analyses.

The prognostic value of VISTA was investigated by Kaplan–Meier analysis using GraphPad Prism 6. Additionally, multivariate survival analysis was performed using the Cox proportional hazards regression model.

## Results

### *VISTA* expression according to characteristics of glioma patients

In total, 57 glioma tissues in the Moroccan cohort (33 men and 24 women) and 667 glioma cases in the TCGA cohort were recruited in the current study. The characteristics of the enrolled Moroccan patients were described in Table 1. The expression profile of *VISTA* was found to be associated with glioma grades (*p* = 0.0422) but not with other characteristics such as the gender (*p* = 0.1295), histological type (*p* = 0.058), smoking status (*p* = 0.5573) and age (*p* = 0.364). However, in the TCGA data set (Table 2), *VISTA* mRNA expression was significantly associated with glioma grades (*p* < 0.0001), histological type (*p* < 0.0001), and molecular subtype (*p* = 0.0002), but not with otherparameters such as gender (*p* = 0.647), age (*p* = 0.4028), Karnofsky score (*p* = 0.055) and IDH mutation status (*p* = 0.0702).

**Table 1:** Expression of *VISTA* depending on patient characteristics.

Variable	Cases (%) (n = 57)	*p* value
**Sex**		
Male	33 (57.9)	
Female	24 (42.1)	0.1295
**Age**		
Children (≤ 18 years)	16 (28)	
Adults (˃ 18 years)	41 (72)	0.364
**WHO grade**		
Low grade (I-II)	27 (47.36)	
High grade (III-IV)	30 (52.63)	0.0422
**Histological type**		
Astrocytomas	44 (77.19)	
Oligodendrogliomas	3 (5.3)	
Ganglioglioma	2 (3.5)	
Ependymomas	7 (12.3)	
Xantoastrocytoma	1 (1.7)	0.058
**Smoking status**		
Yes	10 (17.5)	
No	47 (82.5)	0.5573

**Table 2:** Expression of *VISTA* according to the characteristics of the glioma patient cohort of the TCGA dataset.

Variable	Cases (%) n	*p* value
**Sex**		
Male	327 (49.1)	
Female	339 (50.9)	0.647
**Age**		
Children (≤ 18 years)	3 (0.45)	
Adults (˃ 18 years)	664 (99.55)	0.4028
**WHO grade**		
Low grade (II–III)	515 (77.66)	
High grade (IV)	152 (22.33)	< 0.0001
**Histological type**		
Astrocytoma	245 (36.73)	
Oligoastrocytoma	129 (19.34)	
Oligodendroglioma	293 (43.92)	< 0.0001
**Glioblastoma subtype**		
Mesenchymal	49 (34.3)	
Classical	39 (27.3)	
Neural	26 (18.2)	
Proneural	29 (20.2)	0.0002
**Karnofsky score**		
˃ 80	204 (69.6)	
80–60	84 (28.7)	
˂ 60	5 (1.7)	0.55
**IDH mutation status**		
Yes	135 (23.6)	
No	437 (76.4)	0.0702

### *VISTA* gene expression was upregulated in glioma tissues relative to PBMC of healthy donors

**Figure 1:**
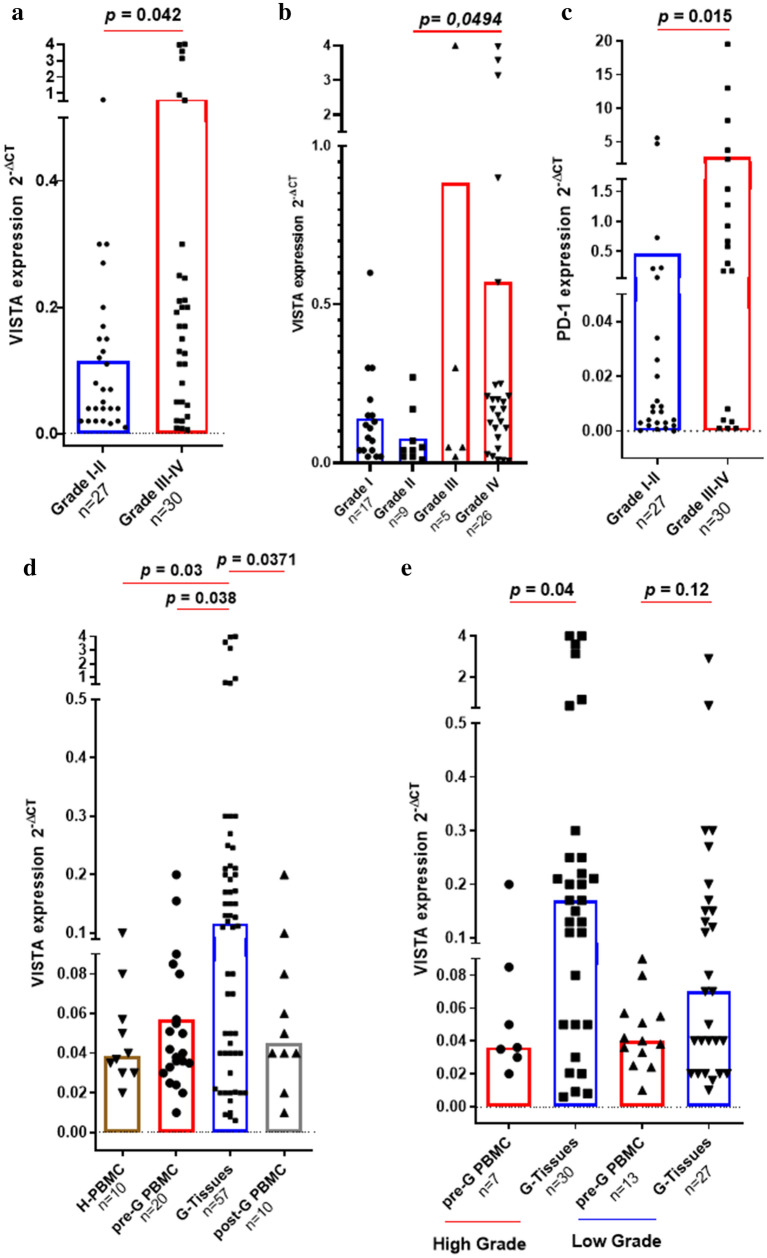
*VISTA* gene expression was upregulated in glioma tissues relative to PBMC of healthy donors (Moroccan cohort). *VISTA* and *PD-1* transcripts expression were performed using RT-PCR analysis. (**a**) *VISTA* gene strongly expressed in grade III–IV compared with grade I–II of glioma patients. (**b**) VISTA mRNA expressionwas significantly increased in advanced glioma (grade IV) compared to grade II. (**c**) *PD-1*was highly expressed in advanced glioma grading (grade III–IV). (**d**) Elevated expression of *VISTA* gene in glioma tissues (G-Tissues) compared to PBMC of healthy donors (H-PBMC) and PBMC of glioma patients before (pre-G PBMC) and after surgery (post-G PBMC). (e) *VISTA* was upregulated in high grade glioma tissues (grade III–IV) relative to PBMC of the same patients. Paired t-test was used to make a statistical comparison of *VISTA* and *PD-1* expression between groups. *p* value less than 0.05 was considered as statistically significant for all statistical analyses.

To evaluate the association between *VISTA* gene expressionand the pathogenesis of glioma, a Moroccan cohort, of 87 samples and 10 PBMCof healthy donors, were analyzed. The levels of expression of *VISTA* were evaluatedby Real-Time RT-PCR. *VISTA* expression was significantly increased in high grade versus lowgrade glioma tissues (*p* = 0.042) (Fig. 1a). VISTA mRNA expression was significantly elevated in glioma grade IV compared to grade II (*p* = 0.0494) (Fig. 1b). Tovalidate VISTAgene expression results observed in our glioma cases, *PD-1* (a known checkpointmolecule) mRNA expression was analyzed in 47 glioma samples. Indeed, as expected, the expression level of *PD-1* was also found to be significantly higher in grades III and IV compared to low grades (I and II) (*p* = 0.015) (Fig. 1c).

To assess whether there is a relationship between *VISTA* expression in the tumor microenvironment versus patient PBMCs, compared with PBMCs of healthy donors, 57 glioma tissuesand 30 PBMC samples of the same glioma patients(20 PBMCs before and 10 after surgery) were analyzed. Expression levels of*VISTA* transcripts were elevated in glioma tissues compared to both PBMCs of healthy donors (*p* = 0.03) and PBMCs from glioma patients before surgery (*p* = 0.012). However, the difference was not significant when compared to PBMCs collected after surgery (*p* = 0.08) (Fig. 1d). When patients were stratified according to glioma grades, we detected a significantly elevated expression of *VISTA *in high-grade glioma tissues than PBMCs collected from the same patients before surgery (*p* = 0.04)*.* In contrast, in low-grade gliomas, no significant difference was observed between *VISTA* expression in tumor tissues versus PBMCs collected again from the same patients before surgery (*p* = 0.12) (Fig. 1e). These observations indicated that the elevated expression levels of *VISTA* were noticed in high versus low glioma grade. This expression was specific to tumor tissues.

### VISTA protein exhibited elevated expression levels inhigh-grade gliomas

**Figure 2:**
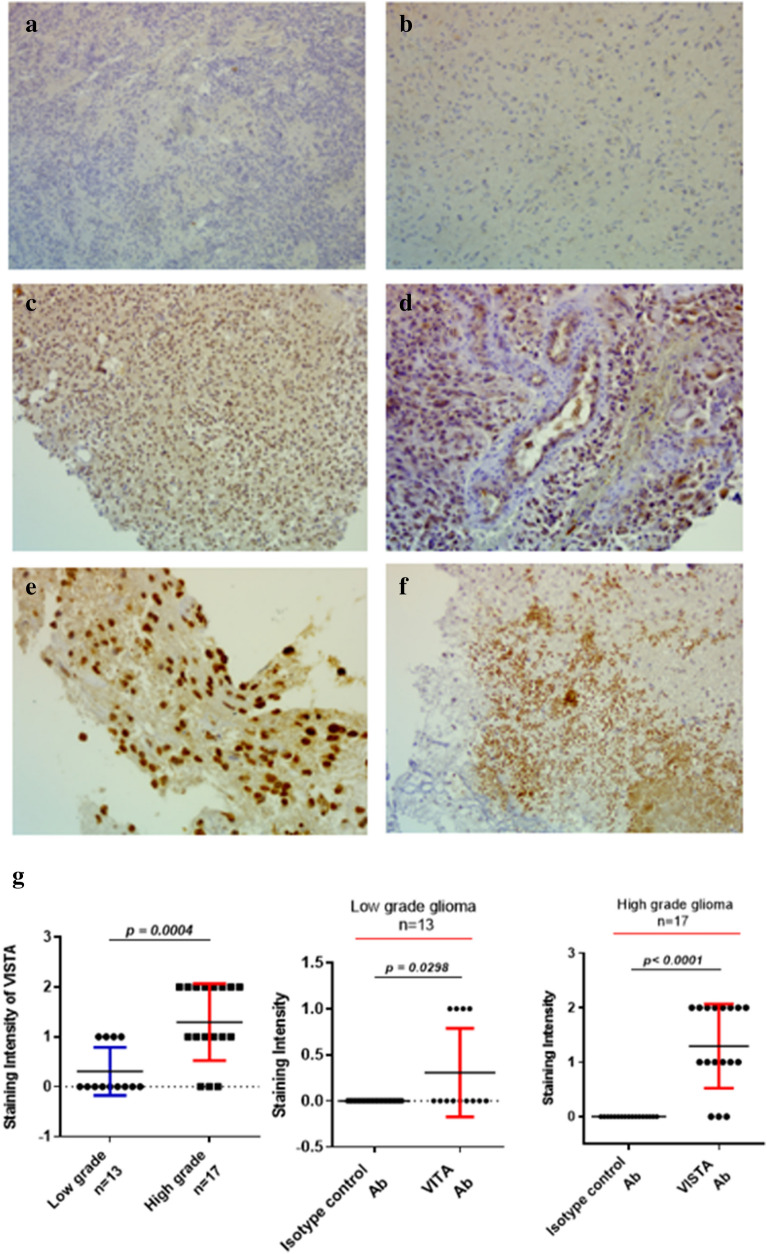
Immunohistochemical staining of VISTA revealed elevated expression levels in high grade gliomas. Representative staining intensity of VISTA protein was detected in human glioma tissues (Moroccan cohort) using immunohistochemistry assay. (**a**) Negative control staining in glioma case with Rabbit IgG Isotype Control (magnification × 20). (**b**) Negative staining of VISTA in low grade glioma (grade I). (**c**) Positive staining of VISTA in high grade glioma (Glioblastoma IV) (magnification × 20). (**d**) Positive staining of VISTA on endothelial cells (magnification × 20). (**e**) Positive staining of VISTA on glioma cells (magnification × 40). (**f**) Positive staining of VISTA on immune cells in a glioblastoma case (magnification × 20). (**g**) Expression of VISTA according to glioma grades. Statistical analysis was performed by using a t-test to compare the expression of VISTA between different grades of glioma patients. *p* value less than 0.05 was considered as statistically significant for all statistical analyses.

In order to corroborate the results of VISTA gene expression obtained in the transcript level (Fig. 1), VISTA protein analysis was performed on 30 glioma cases (13 low grade (I/II) and 17 high grade (III/IV) cases) of the same patient samples by immunohistochemistry assay. The IgG isotype control was assessed in glioma tissue (used as a negativecontrol for VISTA protein expression) (Fig. 2a). Interestingly, VISTAstaining was negative in the majority (9 out of 13 cases, 69.23%) of low-grade gliomas (Fig. 2b). Furthermore, a significant VISTA staining was detected in 82.35% (14 out of 17 cases) ofhigh-grade gliomas (Fig. 2c). VISTA also exhibitedsignificantly higher expression in advanced versus low-grade gliomas (Fig. 2g, p = 0.0004). Notably, VISTAprotein was detected on glioma cells, especially in glioblastoma cases, wheresignificant staining was observed (Fig. 2e). Besides, about 20%of glioma samples showed positive staining of VISTA on immune and endothelialcells (Fig. 2d,f). Altogether, our dataindicate that VISTA was highly expressed at both mRNA and protein levels in patients with high grade compared to lowgrade gliomas.

### *VISTA* transcripts strongly expressed in high glioma grades in the TCGA cohort

**Figure 3:**
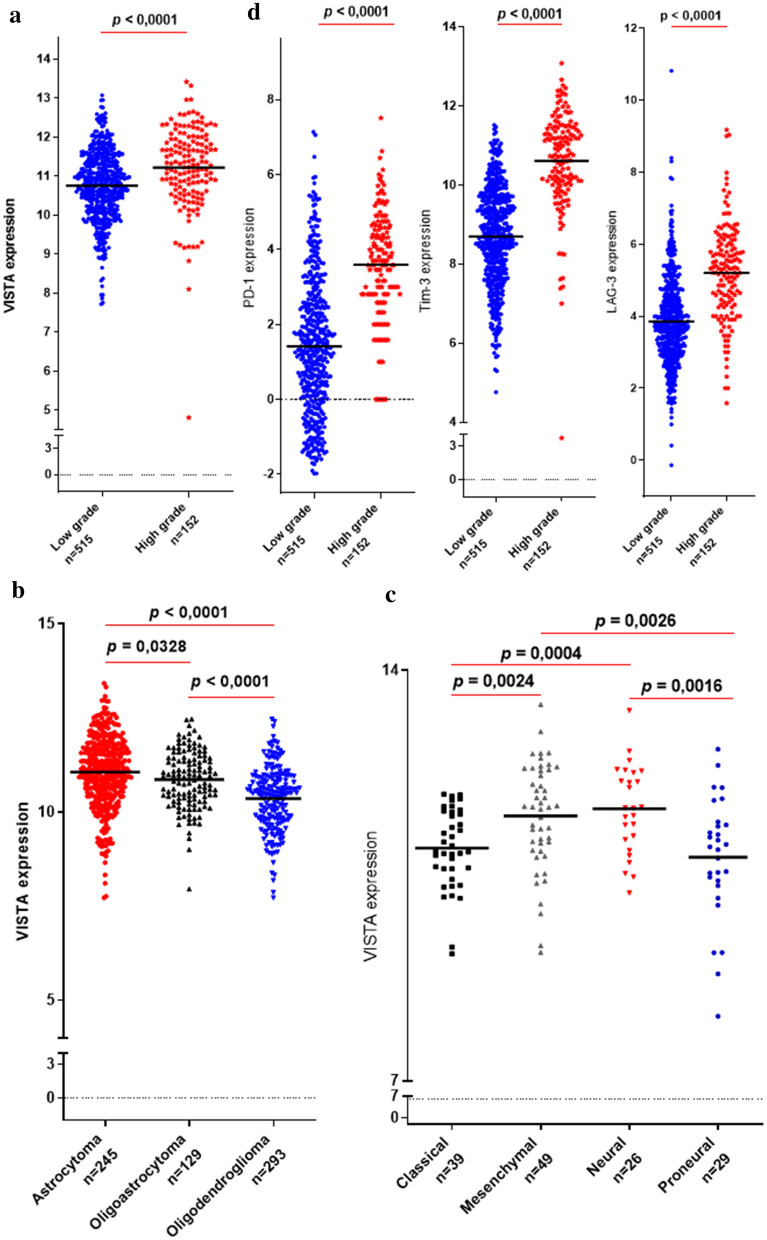
*VISTA* transcripts strongly expressed in high glioma grades in the TCGA cohort and positively correlated with critical immune checkpoint regulators. RNAseq of 667 glioma patients of different grades were analyzed using TCGA dataset. (**a**) *VISTA* mRNA evaluation revealed high expression in advanced gliomas (grade IV). (**b**) Astrocytomas showed elevated expression of *VISTA* compared to oligoastrocytomas and oligodendrogliomas. (**c**) Mesenchymal and Neural glioma subtypes presented high *VISTA* expression in comparison with classical and proneural. (**d**) *PD-1, Tim-3* and *LAG-3* were upregulated in high grade glioma (glioblastoma).T-test was applied to compare gene expression between different grades and groups of glioma patients. *p* value less than 0.05 was considered as statistically significant for all statistical analyses.

To assess the expression of *VISTA* in a distinct cohort, we evaluated the RNA-sequencing data of gliomas from the TCGA database.667 samples were analyzed and graded according to the WHO grading system. Compared to low grade gliomas, glioblastomas present a significantly higher *VISTA* expression (*p* < 0.0001) (Fig. 3a)*.* Further analysisshowed an elevated expression of *VISTA* in Astrocytomacompared to Oligoastrocytoma (*p* = 0.0089) and Oligodendroglioma (*p* < 0.0001) (Fig. 3b). However, Classical andProneural molecularsubtypes revealed lower *VISTA* gene expressioncompared to Neural (*p* = 0.0010 and *p* = 0.0023, respectively*)* (Fig. 3c).

### *VISTA* positively correlated with critical immune checkpoint regulators in glioma patients

The expression pattern of VISTA was compared, using the TCGA dataset, to the expression of three critical immune checkpoints (*Tim-3*, *LAG-3*, *PD-1*) known for being highly expressed in advanced versus low glioma grades^22–24^. Indeed, high glioma grades showed significantly higher *PD-1*, *Tim-3* and *LAG-3* expressions compared to low grades, exhibiting a similar expression profile to *VISTA* (p < 0.0001) (Fig. 3d).

**Figure 4:**
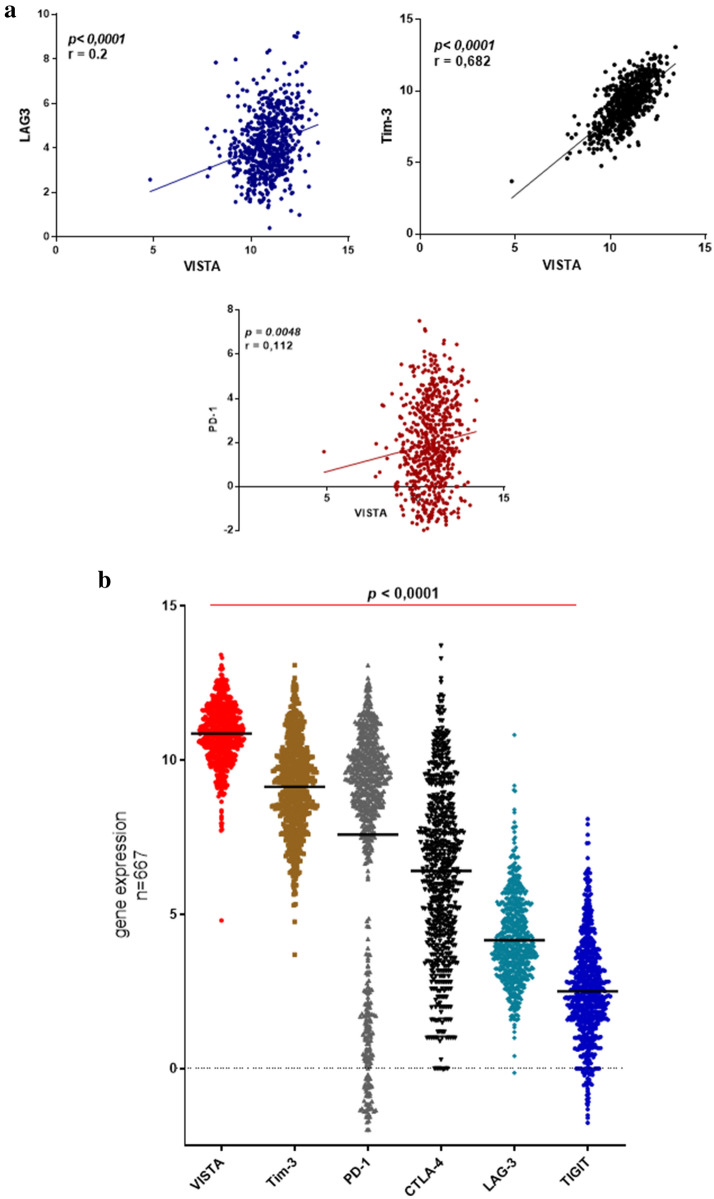
VISTA expression positively correlated with critical immune checkpoint regulators. (**a**) *VISTA* expression was positively correlated with *PD-1*, *Tim-3* and *LAG-3*. (**b**) *VISTA* gene exhibited the highest expression in comparison with other immune checkpoints (*Tim-3, PD-1, CTLA-4, LAG-3, TIGIT*). Spearman correlation test was used to examine the association of relative gene expression levels between VISTA and other immune checkpoints. Also, the one-way ANOVA test was used to determine the statistical significance of gene expression among different groups of glioma patients. *p* value less than 0.05 was considered as statistically significant for all statistical analyses.

A correlation study was conducted between *VISTA* expression and the same immune checkpoints (*PD-1, Tim-3* and *LAG-3*).* VISTA* was positively correlated with *PD-1* (*p* = 0.0048*, r* = 0.112), *Tim-3 *(*p* < 0.0001*, r* = 0.682) and *LAG-3 *(*p* < 0.0001*, r* = 0.2) gene expression (Fig. 4a), suggesting that tumor cells may likely use *VISTA* gene in the same way as these three immune checkpoints to escape the immune system. At last, *VISTA* mRNA expression levels appeared to be the highest in all glioma cases (low and high grade) when compared to those of other critical immune checkpoints, *Tim-3*, *PD-1*, *CTLA-4*, *LAG-3* and *TIGIT* (*p* < 0.0001) (Fig. 4b).

### Anti-tumoral genes were inhibited in glioma microenvironment

**Figure 5:**
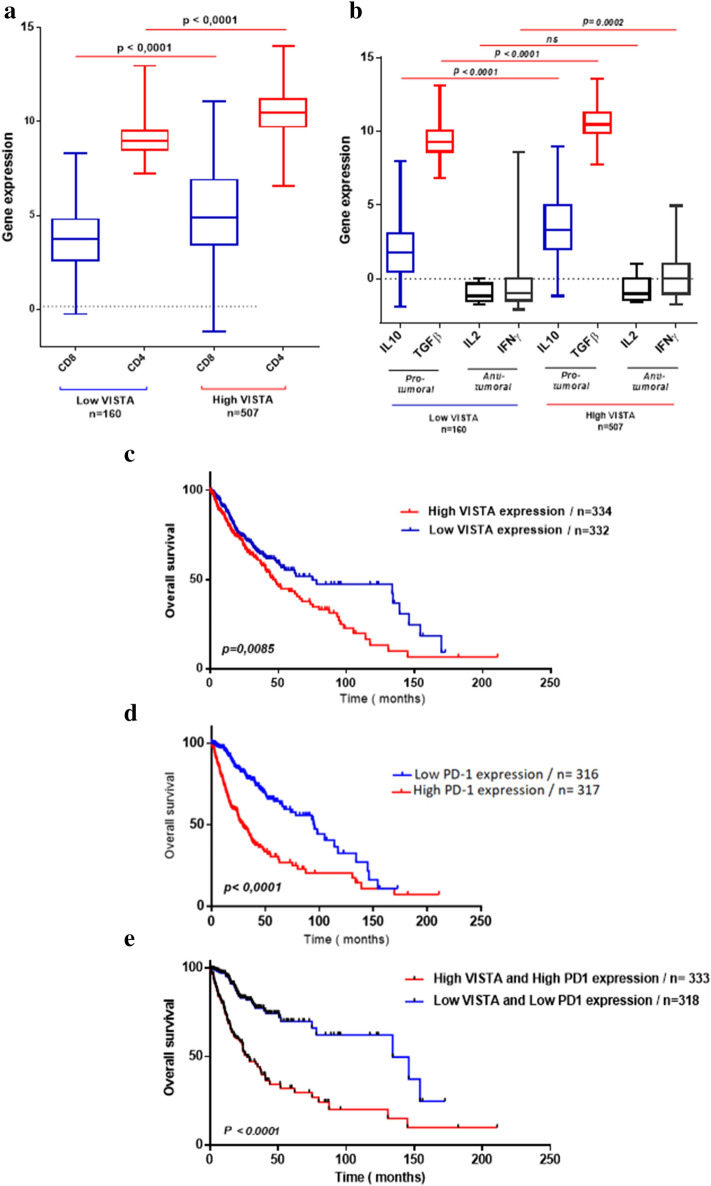
Increased *VISTA* transcripts level associated to a poor prognosis of glioma patients in the TCGA dataset. A binary clustering of patients has been performed, using the median as a cut off for patient stratification: one group with a high expression of *VISTA* and a second one with lower expression. (**a**) *CD4* and *CD8* mRNA expression were both elevated in high versus low *VISTA* expression. (**b**) The immunomodulatory genes (*TGF-β*, *IL-10* and *IFNγ*) showed high expression in glioma patients with higher levels of *VISTA* gene expression. (**c**) High *VISTA* expression levels associated with a bad overall survival. (**d**) Elevated expression of both *VISTA* and *PD-1* correlated with a weak survival. Paired t-test was used to make statistical comparison of gene expression between groups. The prognostic value of *VISTA* gene was investigated by Log-rank test using the Kaplan–Meier plot. *p* value less than 0.05 was considered as statistically significant for all statistical analyses.

To clarify the relationship between *VISTA* expression profile and the occurrence of different immune cell populations in the tumor microenvironment, we have created a binary clustering of patients (TCGA cohort), using the median as a cut off for patient stratification. One group with a high expression of *VISTA* and the second one lower. Then, we assessed the expression of *CD4* and *CD8* genes. *CD4* and *CD8* mRNA expression were both elevated in high versus low *VISTA* expression (*p* < 0.0001). *CD4* mRNA levels were also higher in the elevated *VISTA* gene expression group compared to *CD8 *(*p* < 0.0001) (Fig. 5a), suggesting that CD8 and CD4 cells are present within the tumor microenvironment but with limited effector function because of the higher expression level of *VISTA*. As for T lymphocyte-related cytokines, we assessed the expression of two distinct sets. *TGF-β/IL-10* genes, known to play critical roles in inhibiting CD8 and CD4 T cell functions and *IL-2*/*IFNγ* genes, which are known to boost the anti-tumor immune response^25^. *TGF-β* and *IL-10* gene expression was upregulated in glioma patients who present an elevated expression of *VISTA.* However*, IFNγ*also exhibited a significantly higher expression (*p* = 0.0002)*.* For *IL-2* gene expression, no significant difference has been observed (Fig. 5b).

### Increased *VISTA* transcript levels associated with weak overall patient survival

To examine the impact on patient survival, we evaluated the prognostic value of *VISTA* in the TCGA dataset. Survival data were available for 666 human glioma patients. As showed using Kaplan–Meier curves, patients with lower *VISTA* expression had prolonged survival compared to patients with higher expression of *VISTA *(*p* = 0.0085) (Fig. 5c). In addition, glioma patients with elevated expression of PD-1 showed a poor overall survival (Fig. 5d). Remarkably, patients have increased expression levels of both *VISTA* and *PD-1* showed worse survival compared with those presenting low expression of both genes (*p* < 0.0001) (Fig. 5e). These results indicated that *VISTA* could be considered as a negative prognostic factor in glioma. In order to identify the effects of confounding variables on glioma patients’ survival, the clinical characteristics were selected from the TCGA dataset a variables for multivariate cox regression analysis. Results revealed that in glioma patients (high and low grades), histological type, age and grade were associated with the worse glioma patients’ survival ([HR] = 1.27697, *p* = 0.0132; [HR] = 2.63324, *p* = 2.23 *e *− 09; and [HR] = 2.76359, *p* = 2.67*e *− 08), respectively. However, no significant correlation was found between Sex and VISTA gene expression with clinical outcome (Table S1). Additionally, multivariate cox regression analysis has also been performed on two separate bases, one containing low-grade gliomas and the other high-grade gliomas, in order to evaluate the effect of clinical parameters and VISTA gene expression on patient’s survival. In this case, histological type, grade (II, III), age and Karnofsky score were associated with bad patients’ survival ([HR] = 1.3740, *p* = 0.0201; [HR] = 3.0360, *p* = 6.32e − 05; [HR] = 3.0914, *p* = 2.02e − 05; ([HR] = 2.2554, *p* = 3.57e − 05) (Table S2). Surprisingly, in high grade glioma patients, our results indicated a correlation between patients’ sex and VISTA gene expression with patients’ poor survival ([HR] = 1.981e + 00, *p* = 0.00764 and [HR] = 1.727e + 00, *p* = 0.03192, respectively) (Table S3).

## Discussion

Gliomas are the most frequent and fatal brain tumors in adults^1^. Despite treating glioblastoma patients with conventional therapies such as surgical resection with subsequent radiation and chemotherapy, the prognosis for glioma patients is still poor^2^.

In the last few years, immunotherapy has brought new hope as a potential novel therapeutic approach for glioma patients^26^. However, the majority of glioma patients did not respond to the blockade of usual immune checkpoints pathways (CTLA-4 and PD1/PD-L1)^11–13^.

This has increased our interest in exploring the role of other immune checkpoint molecules, including the recently discovered one, *VISTA*^15,16^.

Thus, the main objective of this work was to investigate the role of *VISTA* in human gliomas. The study revealed that: (1) *VISTA* gene expression was upregulated in high versus low glioma grades, ((2) VISTA protein exhibited elevated expression levels in high-grade glioma, (3) *VISTA* expression positively correlated with other critical immune checkpoint regulators, (4) genes, which are known to be linked to an anti-tumor signature, were inhibited in the glioma microenvironment, (5) elevated *VISTA* transcript levels negatively correlated with the IDH mutation in patients, and (6) high *VISTA* transcript levels associated to weak overall patient survival. To the best of our knowledge. This is the first exploration of the role of *VISTA* in clinically resected glioma tumors. It is also the largest and most comprehensive study describing the expression pattern of VISTA in human glioma samples using two cohorts (TCGA and Moroccan cohort). *VISTA* expression has, however, been investigated in other cancer tissues including colorectal carcinoma^27^, human hepatocellular carcinoma^28^, gastric cancer^21^, human oral squamous cell carcinoma^29^, pancreatic cancer^30^, oesophagal adenocarcinoma^31^ and prostate cancer^21^. In these reports, and consistent with the present work. It has also been shown that *VISTA* expression is upregulated in higher versus lower grades of the disease.

Besides, protein level assessment using immunohistochemistry assay confirmed the VISTA expression pattern initially observed at the mRNA level.

In gastric cancer, using immunohistochemistry in a large cohort of 464 samples and 14 corresponding liver metastases, it was revealed that VISTA expression was observed in tumor and immune cells, but not in non-neoplastic gastric epithelium. Also, this expression varies with tumor progression 21. In the same context using the immunofluorescence method, 28 clinical colorectal cancer specimens were used to evaluate VISTA protein expression showing that VISTA is expressed in normal colorectal samples, in para-tumors and tumors cases, with elevated expression levels in the tumors. However, *VISTA* was revealed to be expressed at high levels in different subsets of myeloid cells in the tumors compared to PBMCs^27^. Otherwise, VISTA expression was suggested to be linked to PD-L1 expression in gastric cancer, suggesting that VISTA cooperates with PD-L1 in the mechanism underlying immune evasion^21^. A recent study revealed that pancreatic tumors with high cytolytic activity have increased the expression of immune checkpoint genes such as *CTLA-4*, *TIGIT*, *TIM-3*, and *VISTA*^32^. In contrast, others have reported that there was no correlation between VISTA and other checkpoint-markers such as PD-L1 and LAG-3 in oesophagal adenocarcinoma cohort, indicating that VISTA might function separately^28^. Chraa et al. reviewed the tumor microenvironment and its infiltration by distinct T lymphocyte subpopulations^33^. This report explained the association of these different subtypes with cancer progression^33,34^. However, treatment with several cytokines, including IFN-γ, IL-2, IL-4, and IL-6, did not raise VISTA expression in tumor cells^35^. Kondo et al. demonstrated that VISTA blockade efficiently converted resting and exhausted T cells into functionally differentiated effector CD8+ T cells, indicating that monotherapy using VISTA might have the ability to enhance multifunctionality of CD8+ T cells in squamous cell carcinoma^16^. Wang et al. showed that in the presence of TGF-ß, VISTA Ig promoted partially the differentiation of iTreg and that this effect could be observed in both murine and human CD4+ T cells^35,36^. Furthermore, VISTA is necessary for the generation of iTreg from naïve T cells, which enhances a microenvironment, which is favorable for the expansion of tumor cells^37^.

Interestingly, blocking VISTA in tumor cells extended mice survival that was inoculated with ovarian cancer cells overexpressing VISTA, although combined therapy using anti-PD-1 and anti-VISTA did not further improve mice survival compared to anti-VISTA treatment alone^38^. Latest studies on CTLA-4 and PD-1 blockade showed an upregulation of VISTA expression in prostate cancer and melanoma treated patients. This observation suggested the importance of considering a potential VISTA blockade for these patients.

In summary, our data revealed a correlation between *VISTA* expression and glioma progression in patients. This study also indicated that *VISTA* is a negative prognostic factor in glioma, and pinpoints *VISTA* as a possible new therapeutic target, particularly in advanced glioma stages.

## Supplementary Information

Supplementary Table S1.

Supplementary Table S2.

Supplementary Table S3.

## Abbreviations

PD-1: Programmed cell death 1
VISTA: V-domain Immunoglobulin suppressor of T cell activation
PDL-1: Programmed death-ligand 1
PDL-2: Programmed death-ligand 2
LAG-3: Lymphocyte-activation gene 3
Tim-3: T-cell immunoglobulin and mucin-domain containing-3
CTLA-4: Cytotoxic T-lymphocyte-associated protein 4
TIGIT: T cell immunoreceptor with Ig and ITIM domains
GBM: Glioblastoma multiforme
TCGA: The Cancer Genome Atlas
PBMC: Peripheral blood mononuclear cell
mRNA: Messenger RNA
WHO: World Health Organization
PCR: Polymerase chain reaction
IL-2: *Interleukin*-*2*
IL-10: *Interleukin*-*10*
IL-6: *Interleukin*-*6*
IL-4: *Interleukin*-*4*
TGFβ: Transforming growth factor β
IFN_γ_: Interferon-gamma
