# Supplementary material for: The immune checkpoint VISTA exhibits high expression levels in human gliomas and associates with a poor prognosis
Source: Sci Rep. 2021 Nov 2;11:21504. doi: 10.1038/s41598-021-00835-0 (PMC8563991; doi:10.1038/s41598-021-00835-0)
Supplement: Supplementary file 1 — Supplementary Table S1. [file 41598_2021_835_MOESM1_ESM.docx]

**Table S1:** Multivariate Cox proportional hazard regression analyses of OS in glioma patients (Low and High Grade).

| **Variable** | **Multivariate Analysis** | |
| --- | --- | --- |
|  | **HR** | ***p value*** |
| **Histological type** | 1.27697 | ***0.0132**** |
| **Grade** | 2.63324 | ***2.23 e-09****** |
| **Age** | 2.76359 | ***2.67e-08****** |
| **Sex** | 0.96422 | *0.7901* |
| **VISTA** | 1.02489 | *0.8643* |

***HR*** hazard ratio*,* ***OS****overall survival.*

*Statistical significance is marked with the star symbol: **p < 0.01, ***p < 0.001, ****p < 0.0001.
