# Supplementary material for: The immune checkpoint VISTA exhibits high expression levels in human gliomas and associates with a poor prognosis
Source: Sci Rep. 2021 Nov 2;11:21504. doi: 10.1038/s41598-021-00835-0 (PMC8563991; doi:10.1038/s41598-021-00835-0)
Supplement: Supplementary file 2 — Supplementary Table S2. [file 41598_2021_835_MOESM2_ESM.docx]

**Table S2:** Multivariate Cox proportional hazard regression analyses of OS in low grade glioma patients.

| **Variable** | **Multivariate Analysis** | |
| --- | --- | --- |
|  | **HR** | ***p value*** |
| **Histological type** | 1.3740 | ***0.0201**** |
| **Grade** | 3.0360 | ***6.32e-05****** |
| **Age** | 3.0914 | ***2.02e-05****** |
| **Sex** | 1.4280 | *0.1378* |
| **History of other malignacy** | 0.4520 | *0.2773* |
| **Karnofsky score** | 2.2554 | ***3.57e-05****** |
| **VISTA** | 0.6912 | *0.1138* |

***HR*** hazard ratio*,* ***OS****overall survival.*

*Statistical significance is marked with the star symbol: **p < 0.01, ***p < 0.001, ****p < 0.0001.
