# Supplementary material for: The immune checkpoint VISTA exhibits high expression levels in human gliomas and associates with a poor prognosis
Source: Sci Rep. 2021 Nov 2;11:21504. doi: 10.1038/s41598-021-00835-0 (PMC8563991; doi:10.1038/s41598-021-00835-0)
Supplement: Supplementary file 3 — Supplementary Table S3. [file 41598_2021_835_MOESM3_ESM.docx]

**Table S3:** Multivariate Cox proportional hazard regression analyses of OS in high grade glioma patients.

| **Variable** | **Multivariate Analysis** | |
| --- | --- | --- |
|  | **HR** | ***p value*** |
| **Age** | 1.981e+00 | ***0.00764***** |
| **Sex** | 9.855e-01 | *0.95740* |
| **Subtype** | 9.446e-01 | *0.61014* |
| **MGMT methylation** | 6.971e-01 | *0.20426* |
| **G CIMP methylation** | 1.175e-07 | *0.99634* |
| **IDH1 statu** | 5.507e-07 | *0.99669* |
| **VISTA** | 1.727e+00 | ***0.03192**** |

***HR*** hazard ratio*,* ***OS****overall survival.*

*Statistical significance is marked with the star symbol: **p < 0.01, ***p < 0.001, ****p < 0.0001.
